# Scaffolds for Cultured Meat on the Basis of Polysaccharide Hydrogels Enriched with Plant-Based Proteins

**DOI:** 10.3390/gels8020094

**Published:** 2022-02-04

**Authors:** Jannis O. Wollschlaeger, Robin Maatz, Franziska B. Albrecht, Annemarie Klatt, Simon Heine, Andreas Blaeser, Petra J. Kluger

**Affiliations:** 1 Reutlingen Research Institute, Reutlingen University, 72762 Reutlingen, Germany; jannis.wollschlaeger@reutlingen-university.de (J.O.W.); franziska.albrecht@reutlingen-university.de (F.B.A.); annemarie.klatt@reutlingen-university.de (A.K.); simon.heine@reutlingen-university.de (S.H.); 2 Institute for BioMedical Printing Technology, Technical University of Darmstadt, 64289 Darmstadt, Germany; maatz@idd.tu-darmstadt.de (R.M.); blaeser@idd.tu-darmstadt.de (A.B.); 3 Centre for Synthetic Biology, Technical University of Darmstadt, 64289 Darmstadt, Germany; 4 School of Applied Chemistry, Reutlingen University, 72762 Reutlingen, Germany

**Keywords:** agarose, gellan, xanthan-locust bean gum blend, cell-laden hydrogels, C2C12 immortalized myoblasts, biofabrication

## Abstract

The world population is growing and alternative ways of satisfying the increasing demand for meat are being explored, such as using animal cells for the fabrication of cultured meat. Edible biomaterials are required as supporting structures. Hence, we chose agarose, gellan and a xanthan-locust bean gum blend (XLB) as support materials with pea and soy protein additives and analyzed them regarding material properties and biocompatibility. We successfully built stable hydrogels containing up to 1% pea or soy protein. Higher amounts of protein resulted in poor handling properties and unstable gels. The gelation temperature range for agarose and gellan blends is between 23–30 °C, but for XLB blends it is above 55 °C. A change in viscosity and a decrease in the swelling behavior was observed in the polysaccharide-protein gels compared to the pure polysaccharide gels. None of the leachates of the investigated materials had cytotoxic effects on the myoblast cell line C2C12. All polysaccharide-protein blends evaluated turned out as potential candidates for cultured meat. For cell-laden gels, the gellan blends were the most suitable in terms of processing and uniform distribution of cells, followed by agarose blends, whereas no stable cell-laden gels could be formed with XLB blends.

## 1. Introduction

Global meat production is on the rise in an effort to satisfy the dramatically increased meat consumption of a growing world population [1]. As large-scale conventional animal agriculture struggles to keep up with the demand, the problems of this industry increase with its size. In recent years, about 73% of worldwide sold antimicrobials are used in animals raised for meat production, increasing the risk of creating antibiotic-resistant bacteria [2]. In addition, over 70% of new infectious diseases in people originate in animals [3]. Furthermore, conventional meat production has a massive negative impact on the environment, considering usage of resources (energy, land, water) and the associated environmental footprint (greenhouse gases, deforestation, biodiversity loss), while animal welfare is also declining [4]. Therefore, more sustainable, safe und animal-friendly alternatives are desperately needed.

Cultured meat or in vitro meat creates the opportunity to produce meat without the need for raising the whole animal [5,6]. The needed cells can be grown in vitro using stem cells, for example, isolated from a small biopsy of an animal. To create a product that is as close as possible to a conventional whole meat cut, not only cells will be needed but a supporting matrix [7,8]. In vivo cells grow and function in a 3D gel-like microenvironment with tissue specific mechanical and biochemical properties, the extracellular matrix (ECM). Scaffolding materials can help to mimic this surrounding to enable cells to grow in a more in vivo state by delivering crucial mechanical cues [9].

The scaffolding material should not only support the cells, but also help to create a product with the desired consistency and appearance. To reproduce all important features of conventional meat, the set of requirements for biomaterials used to produce cultured meat is highly specific. The material should be edible, sustainable, widely available, animal-free, non-toxic, cheap, processable and ideally have none or only a mild taste [7,10,11]. Furthermore, the material can also add nutritional value to the product and improve the mouthfeel and with this, also help to improve customer acceptance [12]. Different types of scaffolds are suitable for cultured meat production and scale-up. While microcarriers can be used to scale up the production of cell mass, fibrous scaffolds can help to induce muscle fiber alignment [10,13,14,15]. Porous scaffolds such as hydrogels could increase nutrient, oxygen and waste transport within constructs. In addition, hydrogels are able to mimic the structure of the ECM and can also be used as bioinks in bioprinting processes, making them ideal candidates for larger cultured meat products such as whole meat cuts [8,10,16,17,18,19,20,21]. However, out of the numerous different hydrogel materials and blends, only a few are edible, widely available and animal-free. Some of these edible, plant-based candidates have already been approved by food safety authorities and are used in the food industry. Within this subset, plant-based and microorganism-produced polysaccharides are groups of biomaterials that include a variety of promising candidates that are also quite well-studied for their cytocompatibility [22,23].

Agar/agarose, gellan gum (gellan) and xanthan-locust bean gum blend (XLB) are approved as food additives and have been successfully used in different studies to investigate the mouse myoblast cell line C2C12 [24,25,26]. Those polysaccharides can form stable, predominantly tasteless hydrogels with tunable properties, but they have hardly any nutritional value [27]. Not only can the nutrient content of the scaffold be increased with the addition of plant proteins, but it can enrich the whole meat product with an extended amino acid profile [28]. Soy protein, pea protein and wheat protein isolates are widely available, cheap and are already used in the food industry to produce vegan alternatives mimicking conventional meat products. While wheat protein could represent yet another interesting source of plant protein, the broad usage of wheat protein, however, is hampered by problems associated with allergies [29] and the exaggerated public perception of wheat protein intolerances [30]. Therefore, soy and pea protein isolates are promising candidates to enrich the formulation of cultured meat scaffolds.

In this study, we investigated the influence of pea and soy protein isolates on the stability and the rheological properties of hydrogels from agarose, gellan and XLB. Furthermore, we examined the cytotoxicity of these blends by testing leachates of the hydrogels on the myoblast model cell line C2C12 and by encapsulating these cells into the hydrogels.

## 2. Results and Discussion

As mentioned earlier, cultured meat products are being developed to address the various environmental issues associated with conventional meat production. Therefore, all materials and processes involved in cultured meat production are required to have minimal emissions and to be energy efficient in order to fulfill the promises made by the cultured meat industry. While all of the biomaterials investigated in this study only have a minor environmental footprint, their potential to provide an eco-friendlier meat alternative also depends on the specific species of the conventional meat product to which they are compared to.

Furthermore, raw material availability must be ensured to be able to produce large quantities at low cost. The polysaccharides (agarose, gellan, xanthan and locust bean gum (LBG)) and protein isolates (pea and soy protein) used here, are already approved as food additives and have various applications in the food industry. The availability of the proteins is significantly higher compared to the polysaccharides. However, in contrast to the protein additives, to create these hydrogels, less than 1% of the basic components are required. The annual worldwide meat production exceeded 300 million tons in the year 2020 [30]. Already, 1–3 million tons of cell-free scaffolds for cultured meat can be manufactured with the current production capacities of the hydrogel precursors (Table 1 annual production of agarose, gellan, xanthan and LBG), using 1% gels. In addition, an increase in the production capacities would likely follow a growing demand.

All relevant information about the requirements for biomaterials used as scaffolds in the cultured meat sector is summarized in Table 1.

**Figure 1:**
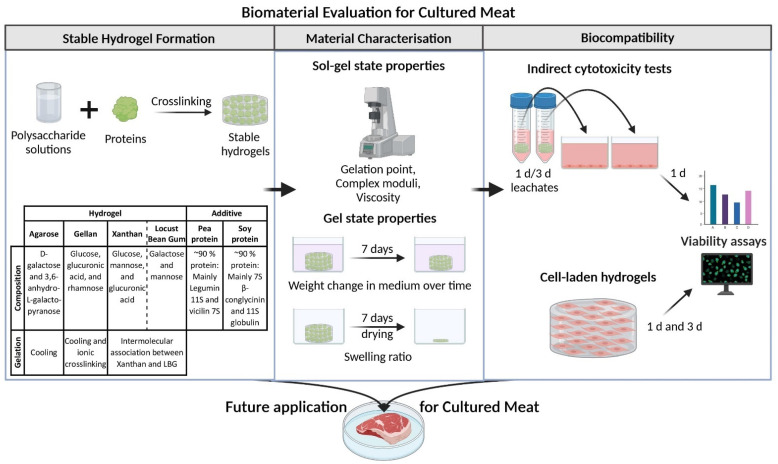
Schematic overview of the biomaterial evaluation in this study, starting with the investigation of stable gel formation of polysaccharide-protein blends and followed by the characterization of the material properties and biocompatibility to find suitable biomaterials for a future application in the cultured meat sector. This figure was created with BioRender.com, (accessed on 28 December 2021).

To increase the nutritional value of the gels and to potentially improve biocompatibility, different protein concentrations of pea and soy protein were tested for stable gel formations. Subsequently, the preselected polysaccharide-protein gel combinations were tested for their material properties in the sol-gel state and gel state and for their biocompatibility by both indirect cytotoxicity testing and viability testing after direct cell-encapsulation (Figure 1).

### 2.1. Characterization of Gel Properties

First, the amount of protein that did not interfere with the preparation and formation of stable gels with agarose, gellan and XLB (gel concentrations: 1% agarose, 1% gellan or 0.5% XLB) was determined. For XLB, a lower concentration was used because at a higher concentration (2% stock solution), the XLB powder was difficult to dissolve, and the resulting solution was very viscous and also difficult to mix with protein solutions. An increase in viscosity at higher concentrations of XLB has been shown previously [70]. The gel stability was assessed visually. The polysaccharides gellan and xanthan-locust bean gum blend undergo various crosslinking mechanisms to form hydrogels from solutions. Cooling plays a role in all of them. Whereas with agarose, this mechanism of self-gelation is solely responsible for gel formation, gellan can additionally be crosslinked with ions including sodium, calcium, magnesium and hydrogen ions [37,71]. Ions are also present in the cell culture medium. Therefore, the gellan gels were additionally crosslinked with the medium to have the same conditions in the cell-free experiments as in the cell culture. In the case of XLB, cooperative interactions between specific segments of xanthan with specific regions of LBG lead to gel formation [72]. 

The addition of salt can actually lower the viscosity of XLB [73,74]. Hydrogels, composed solely of xanthan, usually require acidic compounds such as citric acid or adipic acidic for initiation of xanthan gelation [75]. Creating an acidic environment, however, can affect or even be harmful to cells when they are integrated into such hydrogels. Xanthan-LBG blends can therefore provide a more biocompatible alternative to xanthan alone.

**Figure 2:**
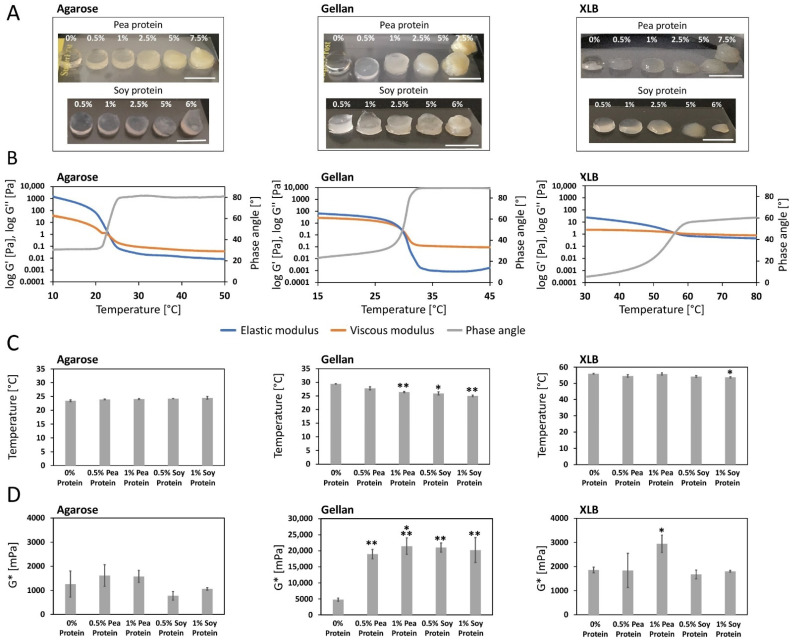
Sol-gel transition properties of agarose, gellan and XLB gels blended with pea or soy protein. (**A**) Gel formation of gels mixed with 0–7.5% pea protein and 0–6% soy protein, respectively. Scale bar: 10 mm. (**B**) Rheological curves of protein-free agarose, gellan and XLB with elastic and viscous modulus and phase angle versus temperature (1.0 Hz frequency and 1 Pa shear stress). (**C**) Temperature-dependent gelation points of polysaccharides blended with 0%, 0.5% and 1% pea or soy protein. (*n* = 3, two-sided t student test * *p* < 0.05, ** *p* < 0.01, respectively to 0% values). (**D**) Complex modulus at gelation points of gels blended with 0%, 0.5% and 1% pea or soy protein (*n* = 3, two-sided t student test * *p* < 0.05, ** *p* < 0.01, *** *p* < 0.001, respectively to 0% values). Error bars represent standard deviations.

In the literature, soy gels with up to 20% protein content have been produced [76]. However, soy protein isolates are not easily dissolved in water unless the pH of the solvent is increased or decreased from a pH of 7 [77]. Accordingly, stock solutions of 12% soy protein and 15% pea protein were prepared and mixed with the three polysaccharides to obtain protein blended gels containing up to 6% soy or 7.5% pea protein. The manufacturing of stable and uniform hydrogels was possible for agarose up to 2.5% protein supplementation. It was still possible to form gels with higher protein concentrations, but they were less dimensionally stable (Figure 2A). The increasing amount of protein may make it more difficult for the single agarose chains to agglomerate during the gelling process, leading to less stable gels. A similar effect was observed in another study when agarose was mixed with xanthan. The xanthan chains interfered with the agglomeration of the agarose chains by hindering them spatially [78]. Uniform gellan-protein blended hydrogels could only be casted using a maximum of 1% of protein. At higher concentrations, gellan began to gel more quickly during manufacturing, resulting in less uniform gels with rising protein concentration (Figure 2A). 

An explanation for this may be the binding of positively charged amino acid side chains of the plant proteins to the negatively charged structures of gellan [79,80]. The ionic bonding of cations between the gellan chains could thereby be complicated, and thus would have an influence on gel stability. The observed faster gelation times at higher protein concentration may be explained by a faster cooling process. XLB gels were only stable up to 1% protein supplementation. With higher protein concentrations, mixing became more complicated and resulted in heterogeneous gels that fell apart (Figure 2A). Since the xanthan chains are also negatively charged, the positively charged protein side chains can also bind here and thus interfere with the binding to LBG chains [80]. 

Based on these results, preselected protein concentrations of 0.5% and 1% were used to perform all further tests.

#### 2.1.1. Impact of Protein Supplementation on Sol-Gel Transition

The viscoelastic properties of the hydrogels and their blends were determined by oscillatory rheological measurements. First, the gelation point of the three polysaccharide-based hydrogels ranging from 23.5 °C (agarose) to 29.4 °C (gellan) and 55.9 °C (XLB) were analyzed (Figure 2B,C). It is worth mentioning that the high-sol-gel transition temperature of XLB generally limits its applicability in the field of biofabrication, e.g., 3D-bioprinting [81], where cells are mixed with the hydrogel precursor prior to gelation [81]. Nevertheless, the material may still be used as a cellular building block to mechanically enhance the overall structure of biofabricated tissue constructs or as a niche that recruits surrounding cells during tissue maturation.

Next, the impact of protein supplementation was investigated. Differences in the gelation point can be observed for all investigated materials. However, both agarose and XLB protein supplementation only result in slight shifts in the sol-gel transition point in the order of +/− 4%. Interestingly, a significant shift (−5% to −15%) of the gelling point towards lower temperatures can be observed in the investigated gellan samples. For instance, supplementation with 0.5% soy protein reduced the sol-gel transition to 25.9 °C. A closer look at the rheological raw data (Figure S1) elucidates this effect. The shift of the gelation point, which is defined as the cross point of the measured elastic and viscous modulus, can be attributed to an overproportioned increase in the elastic modulus compared to its viscous counterpart at higher temperatures. 

In general, temperature-dependent gelation of polysaccharides occurs due to the enhanced interactions of carbohydrate molecules with each other, at decreasing temperatures, resulting in crosslinking [79]. Proteins are known to form additional interactions with carbohydrate molecules [82,83,84,85]. It is conceivable that at the gelation temperature of gellan with 0% protein (29.4 °C), there is a lower proportion of cross-linked polysaccharide chains in the protein blends compared to gellan without protein, which means that no gelation point is yet visible in the blends. By lowering the temperature, the proportion of crosslinking increases further, and the gelation point is subsequently reached. Moreover, it is possible that the proteins cause a steric hindrance of the polysaccharide crosslinking, whereby the required proportion of crosslinks for the gelation point is only reached at lower temperatures. The data indicate that the elastic properties of gellan hydrogels are more strongly affected by protein supplementation than in the compared agarose and XLB gels. The results of the second experiment, which contrasts the elastic properties of the gels (Figure 2D), support this finding.

#### 2.1.2. Impact of Protein Supplementation on the Complex Shear Modulus

To compare the mechanical properties of the hydrogels, their complex shear moduli were rheologically measured. Since the materials gel at different temperatures (as described previously), the complex shear modulus was assessed at the gelation point to enhance the comparability of the study. Pure agarose (1260 mPa) and XLB (1860 mPa) delivered comparable results. In contrast to these, the shear modulus of pure gellan at the gelling point was increased by more than the factor of two (4813 mPa; Figure 2D). Interestingly, protein supplementation strongly affected the shear moduli of the studied materials. Shifts of 39% to 58% compared to the pure material were observed in agarose and XLB, yet no clear trend was visible. For instance, 1% pea protein supplementation increased the shear modulus of XLB to 2944 mPa, whereas 1% soy protein resulted in almost unaltered values (1804 mPa) compared to the pure material. 

Protein-polysaccharide interactions are associated with the complex formation of micro- and macro-structures, which are accompanied by a change in rheological properties. These depend on the protein and the polysaccharides that were mixed [82,83,84,85] and is driven mainly by forces of electrostatic interactions [86]. The structure of a protein is determined by its amino acid composition as well as its environment (e.g., pH and ionic strength) [87]. In addition, proteins show a different number of exposed charged amino acids [88]. Polysaccharides can also carry a different number of charged groups, which is associated with a different number of intra- and inter-molecular bonds and also depends on the environmental conditions. Furthermore, polysaccharides form different structures when they gel [89]. The significantly higher shear modulus in XLB 1% pea (2944 mPa) can be explained by the fact that XLB carries a higher number of negative charges than gellan and agarose. 

In addition, under the present conditions, there may be a higher degree of intermolecular interactions with pea and thus the protein-polysaccharide interactions lead to higher strength. It can be seen that 1% soy (1804 mPa) does not exhibit this behavior, which could be explained by a lower level of protein-polysaccharide interactions between soy and XLB, in comparison to pea and XLB. Nevertheless, the above-mentioned circumstances of protein-polysaccharide interactions make it difficult to compare the rheological behavior of polysaccharide-protein blends. Further, the results are in a protein concentration range for which a trend is not yet apparent, but which has been shown to be suitable, based on our previously acquired results (Figure 2A). Due to this, we consider it less relevant to choose a larger concentration range.

With an increase of approximately 400%, the shear modulus of gellan exhibits the strongest alteration upon protein addition. For instance, supplementation of 1% pea protein yields a shear modulus of 21,451 mPa. Nevertheless, it should be regarded as an artifact of the shifted gelation point that was previously described (see Section 2.1.1). Correcting this effect by comparing the complex modules of protein-added gellan formulations at a similar temperature (e.g., at 27 °C), the values of the complex modules are converging (0% = 19,250 mPa, 1% pea = 18,020 mPa and 1% soy protein = 18,800 mPa). In summary, the comparison of different gel blends, that exhibit strongly varying sol-gel transition points, is challenging. In addition, it should be noted that the measured shear modulus at the gelling point only provides valuable information for the processability of the hydrogel formulation, e.g., using 3D-bioprinting, but does not directly represent the mechanical properties of the material.

#### 2.1.3. Impact of Protein Supplementation on the Homogeneity of the Polysaccharide Solution

**Figure 3:**
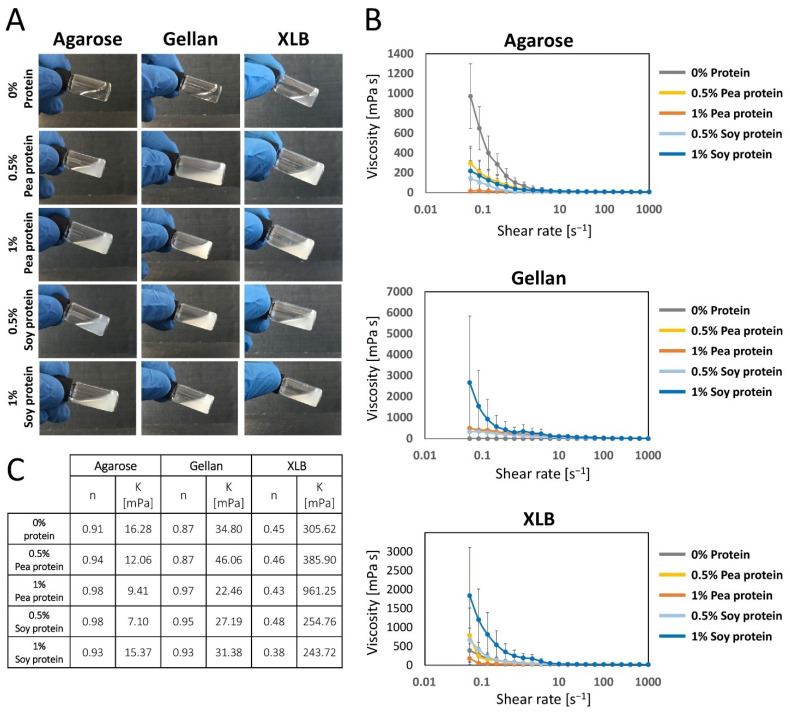
Sol state properties of agarose, gellan and XLB gels blended with 0%, 0.5% and 1% of pea or soy protein. (**A**) Liquid form of the different polysaccharide blends. (**B**) Viscosity over log_10_ (shear rate) curves of the different polysaccharide blends (agarose and gellan at 37 °C, and XLB at 65 °C). Error bars represent standard deviations. (**C**) Determined flow exponent (n)- and consistency factor (K)-value of the different gels.

Agarose and gellan are transparent in the liquid state and become cloudy white after adding either soy or pea proteins. XLB is already slightly opaque without protein and becomes even cloudier after the addition of protein solutions (Figure 3A). All liquids showed a uniform coloration which indicated a homogeneous distribution of the proteins. This allowed for good further processing and continued uniform protein distribution in the solid hydrogels.

#### 2.1.4. Impact of Protein Supplementation on the Complex Shear Viscosity

Assessment of the flow behavior of the studied hydrogel formulation is an important aspect when it comes to their automated processing in the future, e.g., using 3D bioprinting technology. This requires knowledge of the zero-shear viscosity as well as the shear thinning and shear thickening behavior. Therefore, the viscosity of the materials was measured over a wide shear rate range (0.1–1000 s^−1^) at temperatures above the gelation point. All gels exhibit non-newtonian (shear thinning) flow behavior and present a broad viscosity spectrum (Figure 3B). To quantitatively describe the rheological behavior of the gels, we determined the consistency factor (K) and the flow exponent (n) using the Ostwald-de Waele model, as previously described [90]. The consistency factor allows quantitative comparison of the different zero-shear viscosities of the materials, while the flow exponent indicates the degree of shear thinning. 

The n and K values are concentration dependent, which has to be considered for the comparison of the hydrogels when it comes to their suitability in additive manufacturing [91]. Due to specific aspects that have emerged from our study (Section 2.1 and Section 2.2), the concentration range for measuring shear viscosity was chosen and is thus limited to this area. Considering the flow exponent in the range of the chosen concentrations (Section 2.1 and Section 2.2), the investigated materials extend over a broad zero-shear viscosity spectrum ranging from 16 mPas (agarose) to over 300 mPas (XLB). In general, these values represent a very good range for future post-processing using drop-on-demand 3D-bioprinting technology [90]. 

However, the materials strongly differ in their shear thinning behavior, as indicated by the strongly varying flow exponent ranging from 0.91 (agarose) to 0.87 (gellan) and 0.45 (XLB) (Figure 3C). In this regard, XLB may be of high interest for future processing using drop-on-demand 3D-bioprinting technology. Its strongly expressed shear thinning behavior reduces potential shear stress during printing, while its zero-shear viscosity is high enough to maintain structural integration and good shape fidelity of the printing structure. Nonetheless, from a rheological point of view, the other two gels, agarose and gellan, are also well suited for different biofabrication processes, as evidenced by their low zero-shear viscosity and their broad application spectra. 

For all three materials, a slight change in the shear viscosity and shear thinning behavior is observed when pea and soy protein components are added (Figure 3C). However, no consistent trend can be seen in the influence of the protein addition on the consistency factor. Instead, the results indicate that the flow behavior of the gels due to the addition of the proteins varies depending on the matrix material. Therefore, we conclude that the added proteins interact with polysaccharide chains [82,83,84,85]. Adding proteins to a polysaccharide solution can generally increase the shear viscosity due to a higher stability of the protein-carbohydrate interactions. This would also lead to a higher zero-shear viscosity. For all three materials, a slight change in the shear viscosity and shear thinning behavior is observed when pea and soy protein components are added (Figure 3C). However, compared to the base hydrogel matrix, protein supplementation has only little effect on the rheological properties and only plays a minor role in altering the rheological process window. 

Unexpectedly, high shear thinning is shown by XLB (0.45), which could be of great interest to the 3D bioprinting process. Especially there, the shear thinning behavior leads to a favorable property that has a positive effect on the printing process, which is characterized by the fact that the material is highly thinned during flow in the nozzle system and shows high dimensional stability and a low tendency to spread after flowing out [83,84,85].

#### 2.1.5. Impact of Protein Supplementation on Swelling Properties of the Hydrogels

**Figure 4:**
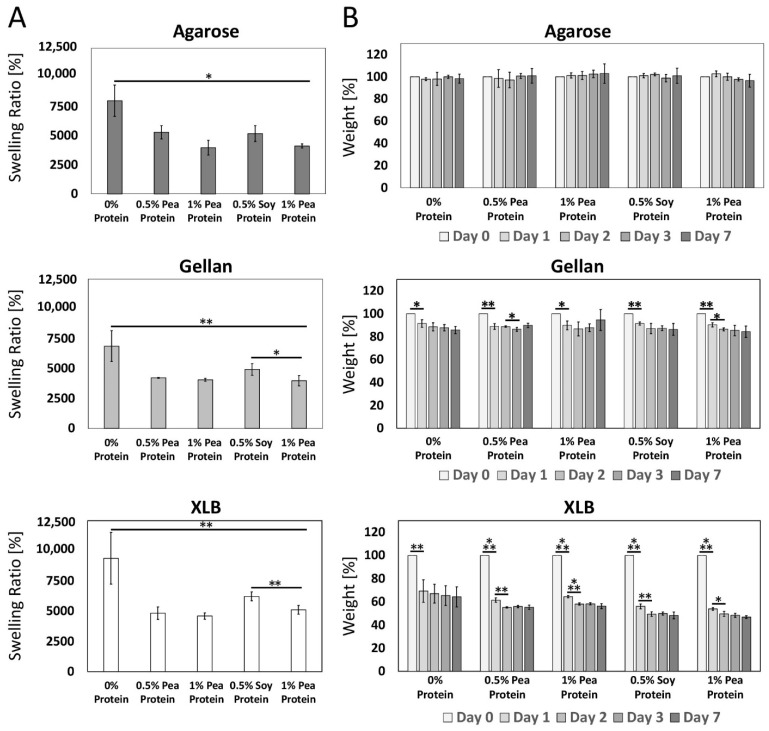
Swelling properties of agarose, gellan and XLB gels blended with 0%, 0.5% and 1% of pea or soy protein in gel state. (**A**) Swelling ratio of different gels (*n* = 4, two-sided t student test * *p* < 0.05, ** *p* < 0.01). (**B**) Weight change of different hydrogels over seven days in medium (*n* = 4, two-sided t student test * *p* < 0.05, ** *p* < 0.01, *** *p* < 0.001). Error bars represent standard deviations.

After characterization of the sol and sol-gel properties of the different hydrogels, the swelling ratios of the different hydrogels with or without protein were determined. In addition, the swelling behavior in cell culture medium was investigated by analyzing weight changes over seven days. For all three polysaccharides, the swelling ratios were significantly higher for the protein-free gels compared to the protein-supplemented gels. Within the protein-containing gels, at least for the soy gels, a decrease in the swelling ratio with increasing protein content was observed (not significantly for agarose; Figure 4A). This increase makes sense if the dry weight is considered, since the proteins increase the dry weight but not the water absorption capacity of the liquid in comparison to the respective polysaccharide. The swelling and release behavior of hydrogels plays an especially important role in drug delivery research. The swelling and release behavior of hydrogels depend, amongst others, on the hydrogel material, pH value, crosslinking process, temperature and the storage fluid [92].

The initial weights for the swelling behavior measurements of the 100 µL gels were determined for all polysaccharides after incubating them 15 min in 1 mL media. This allowed for a better comparability between the additional ion crosslinked gellan and the other gels. The agarose gels showed no change in weight over the seven days in medium (Figure 4B). This behavior of agarose was shown before, even over a longer period [93]. Accordingly, neither the proteins nor agarose components were released into the medium to any extent. In contrast, gellan gels had a weight loss of around 10% within the first day, followed by another 5% weight loss up to day seven (Figure 4B). Since this decrease is similar in samples with or without protein supplementation, it is more likely to be due to the release or shrinking of gellan structures and less to protein release. The release of gellan chains was also previously described, with a weight loss between 5–15% over a period of 168 days [94]. The XLB gels showed the largest weight loss of around 30–40% on the first day. In addition, another decrease could still be observed over the next few days; however, not at such a high rate (Figure 4B). For the protein-free gels, weight reduction was not as strong with 64.3 ± 8.7% of the original weight by day seven compared to the protein-containing gels (0.5% pea protein: 55.3 ± 1.9%; 1% pea protein: 56.3 ± 2.2%; 0.5% soy protein: 48.3 ± 2.9%; 1% soy protein: 46.8 ± 1.2%). This may indicate that more proteins are released from XLB than polysaccharide chains. But further testing is required to confirm that there is a release of proteins from XLB gels. Subsequently, this can be addressed by conducting release experiments, in which the protein concentrations in the supernatant can be determined [95].

### 2.2. Biocompatibility of the Biomaterials

#### 2.2.1. Impact of Biomaterial Leachates on Cell Viability and Metabolic Activity

**Figure 5:**
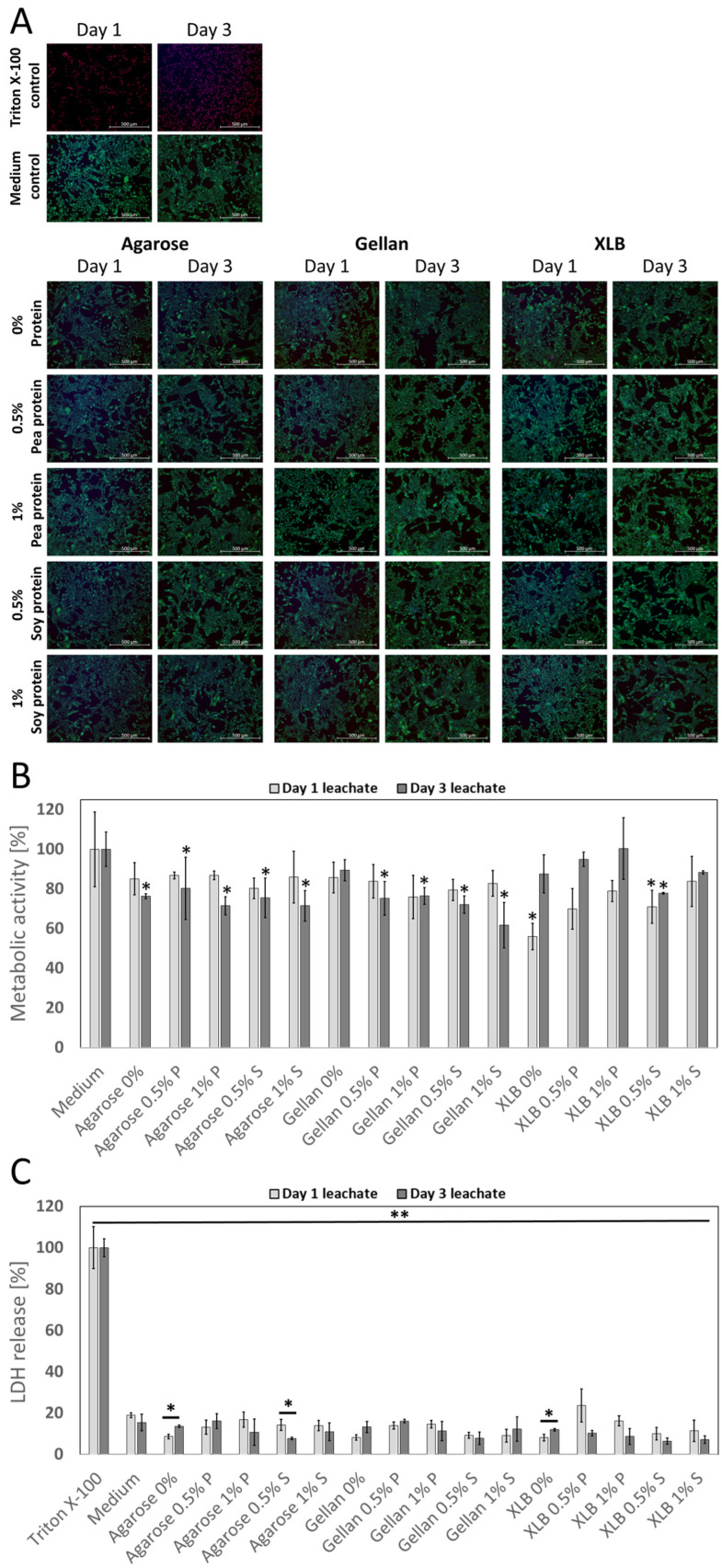
Biocompatibility testing using leachates (day 1 and day 3) of agarose, gellan and XLB gels blended with 0%, 0.5% and 1% of pea or soy protein with one day incubation on C2C12 cells. (**A**) Live/Dead-staining of C2C12 cell. Green: viable cells; red: dead cells; blue: DNA/cell nuclei. Scale bar: 500 µm. (**B**) Metabolic activity of C2C12 after one day treatment with different leachates (day 1 and day 3) relative to medium control (medium treated same as leachates) (*n* = 3, two-sided t student test * *p* < 0.05 respectively to medium control). (**C**) LDH release of C2C12 cells after one day treatment with different leachates (day 1 and day 3) relative to 0.1% TritonX-100 treated cells. (*n* = 3, two-sided t student test * *p* < 0.05, ** *p* < 0.01). Error bars represent standard deviations.

Day 1 and day 3 leachates of the different polysaccharide-protein hydrogel blends were tested for a one-day incubation period on C2C12 cells to observe their effects on cell viability and metabolic activity. The Live/Dead-stainings of the C2C12 cells show no differences for all samples compared to the medium control (Figure 5A), with only a few dead cells, whereas the TritonX-100 control shows only dead cells. Metabolic activity decreases in all leachates but decreases only significantly in leachates from XLB (day 1), XLB with 0.5% soy protein (day 1 and day 3), leachates from agarose (day 3), and leachates from gellan-protein blends (day 3), compared to medium controls (Figure 5B). However, the LDH release, which is released from cells only after cell death, is significantly lower for all samples compared to TritonX-100 controls and not significantly higher than the medium controls (Figure 5C). 

One possible explanation for the decreased metabolic activity, which occurs primarily in the day 3 leachates of agarose and gellan samples, can be a nonspecific binding of medium components to the polysaccharide structures or embedded proteins. In regenerative medicine, such mechanisms are mainly investigated for the immobilization and release of growth factors [96]. Since fetal calf serum (FCS) is used in the medium, which contains many different components such as growth factors, vitamins, peptides, proteins and more [97], it is difficult to identify specific components that may influence cellular activity. This effect may be less pronounced with XLB leachates, considering the results of the swelling test. They intrinsically lose weight over time and thus may bind fewer other substances. 

However, this phenomenon may also be a problem in the subsequent application of cell-laden gels since the availability of the medium components for the cells may also be limited there. It would be interesting to see if this phenomenon also appears in FCS-free media, since all resources for cultured meat applications should be animal-free. FCS-free or serum-free media for mammalian cell culture and cultured meat research are currently extensively investigated, using, amongst others, recombinant growth factors and proteins [98,99,100,101]. Considering all the results of the biocompatibility tests (Live/Dead-staining, LDH assay, metabolic activity assay), however, there is no significant toxic influence of any of the used biomaterials or blends.

#### 2.2.2. Impact of Cell Encapsulation on Cell Viability

**Figure 6:**
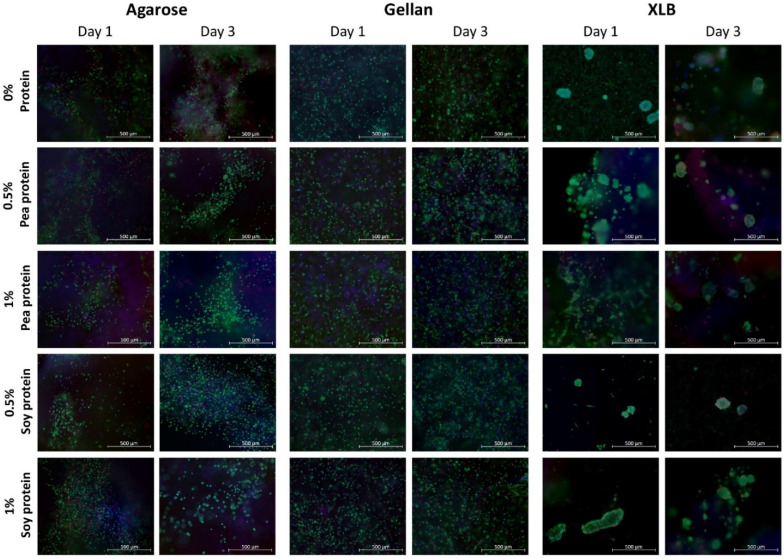
Live/Dead-staining of encapsulated C2C12 cells in agarose, gellan and XLB protein blended hydrogels (0%, 0.5% and 1% pea or soy protein) after one day and three days of culture. Green: viable cells; red: dead cells; blue: DNA/cell nuclei. Scale bar: 500 µm.

To investigate the viability of the cells in direct contact with the biomaterials, in addition to indirect toxicity, cells were encapsulated directly into the different hydrogels. The liquid polysaccharide solutions were tempered to 37 °C before mixing with the C2C12 cells to avoid heat-induced cell death. At this temperature, however, the agarose gels were already too viscous and started gelling while mixing. Subsequently, the Live/Dead-staining showed a heterogeneous distribution of single and rounded cells and a formation of cell clusters within all agarose hydrogels (Figure 6). Most of the cells survived this procedure and were still alive after one day and three days in culture medium. It was easy to mix the liquid gellan-protein blends, as gellan remained liquid. In addition, the cell distribution within the gels was homogeneous and the cells showed a rounded morphology. The proportion of living cells was also high after one day and three days of culture (Figure 6). 

In contrast, mixing of cells with the XLB-protein blends was challenging, due to the high gelation temperature of the gels of around 55 °C (Figure 2C); the material already gelled before the mixing procedure with cells (37 °C) started. Pouring of gels within the preparation molds was still possible, but no stable gels were formed after the addition of medium (Figure S2). This caused the release of cells from the hydrogels. The released cells attached to each other and formed cell aggregates or were present as single cells (Figure 6). Since XLB is already in the gel state and the viscosity of agarose has already increased at 37 °C (Figure 2B,C), these are not optimal conditions for cellular encapsulation. Higher temperatures of the hydrogel solutions can solve this problem, but also increase cell death [102]. Since this is not desirable, other mixing techniques can also be helpful to improve cellular distribution within high viscous gels [103]. For example, in the mixing technique used in this study, gellan with and without protein is the best candidate to encapsulate cells and to build up a 3D cell environment. 

No difference was observed between the protein blended and the pure polysaccharide gels. Thus, it cannot be shown that the proteins had a supporting effect on cell adhesion or cell growth within the three days. The more the natural environment of the cells, such as the ECM, is mimicked, the better the cells are supported by the biomaterials and show improved proliferation [104]. Agarose, gellan and XLB can give a supportive matrix environment for the cells, but do not support cell adhesion per se without being chemically or physically modified, e.g., crosslinking with peptides or RGD sequences [105,106,107]. The plant-based proteins can potentially support cell adhesion, which can also be improved by aforementioned modifications [21,108,109]. Despite all this, the spatial spread of the cells may also be limited by the surrounding matrix, even if the biomaterials can support cell growth as a substrate [108,109,110,111].

To investigate the supportive properties of the biomaterials more in detail, the next step could be to perform specific stainings of proteins involved in cell adhesion, such as vinculin. Furthermore, a phalloidin-actin staining could provide a better insight into the predominant cell morphology [112]. In future studies, it would be interesting to determine if the cells stay viable in the gel during a longer cultivation period and if the protein supplements have an influence on cellular growth, cell morphology and differentiation.

## 3. Conclusions

Some main aspects can be derived from the results concerning the application of the polysaccharide-protein blended hydrogels for the cultured meat sector. First, it is possible to make protein blends (containing up to 1% of pea and soy protein) with all polysaccharides to increase the nutritional value without significantly affecting either the mechanical properties of the gels or cell viability within the gels. For agarose and gellan, even higher protein concentrations are possible, although with a decrease in hydrogel stability. These two candidates also show good gel stability over seven days in medium, whereas XLB significantly loses weight over time. Sustained hydrogel stability is useful to investigate whether the addition of protein promotes cell adhesion, cell growth and myogenic differentiation during prolonged cell culture. In addition, hydrogels must be non-toxic for muscle cells, which was confirmed for all the used biomaterials. Finally, there must be the possibility of encapsulating the cells homogeneously in the hydrogel solution while maintaining their viability. 

All requirements were most suitably fulfilled by gellan. However, for applications where an uneven cellular distribution is not an issue, cell-laden gels could also be prepared with agarose. For XLB, all cell-laden gels broke apart after rinsing with medium. The collective material properties are summarized and assessed in Table 2. Considering all these findings, gellan is best suited for cell encapsulation with an application in the cultured meat sector. Nevertheless, agarose performs well with optimization needed for cell-containing gel assembly. Only XLB showed some processing difficulties due to the comparatively high processing temperature. Due to its biocompatibility, it is still conceivable to find another use for cultured meat applications.

## 4. Materials and Methods

### 4.1. Hydrogel Preparation and Characterizations

#### 4.1.1. Preparation of Hydrogels

Three polysaccharides and two protein isolates were used for preparation of hydrogels. As polysaccharides agarose (No. BP160-100, Fisher BioReagents^TM^, Waltham, MA, USA), gellan (Kelcogel^®^ F, CP Kelco Germany GmbH, Großenborde, Germany) and xanthan-locust bean gum blend (XLB; Kelgum^®^, CP Kelco Germany GmbH) were mixed with different stock solutions of pea protein isolate (2% or 15% (*w/v*) PPI; Empro E 86 HV, Emsland Group, Emlichheim, Germany) and soy protein isolate (2% or 12% (*w/v*) SPI; No. 905456, MP Biomedicals, Solon, OH, USA). 2% (*w/v*) stock solutions were used for agarose and gellan and 1% (*w/v*) for XLB. All three polysaccharide solutions were boiled in the microwave until solving. Then, they were mixed with a prewarmed mix (37 °C for agarose and gellan; 65 °C for XLB) containing distilled H_2_O, protein solution and 150 µL DMEM high glucose (DMEMhg; No. L0101-500, Biowest, Nuaillé, France). The tested combinations and mixture ratios are shown in Table 3. After mixing, the warm gel solution was transferred into a 1 mL syringe (Injekt^®^-F, B. Braun, Melsungen, Germany). Afterwards, 100 µL was given into in-house molds (7 mm diameter, 7 mm height), previously put into 24 well-plates (No. 662 160, Greiner Bio-One GmbH, Frickenhausen, Germany), and cooled down for 15 min at RT for gelation. The molds were used to ensure that the liquid polysaccharide solutions took on a reproducible, cylindrical shape during the gelation process. Gellan gels were additionally crosslinked after cooling by adding 1 mL DMEMhg for 15 min. The hydrogels were evaluated macroscopically for their form stability. The gels with 0%, 0.5% and 1% protein concentration prepared with 2% protein solution were characterized further for their rheological, mechanical and cytotoxic properties.

#### 4.1.2. Rheological Properties of Hydrogels

For all rheological measurements, a rotary oscillating rheometer (Kinexus, Malvern Panalytical, Worcestershire, UK) was used with a 1° cone-plate and 60 mm diameter geometry. Shear viscosity was measured in a shear rate range from 0.1 s^−1^ to 1000 s^−1^ in decadal steps. Samples were heated up to a liquid state before measurement and then measured at a constant temperature (gellan and agarose: 37 °C, XLB: 65 °C).

The samples were transferred to their liquid state to measure the temperature-dependent sol-gel transitions. They were measured by oscillation at a constant frequency of 1 Hz and shear stress of 1 Pa. Measurements were performed at a material-dependent temperature range (gellan: 45–15 °C, XLB: 65–30 °C and agarose: 50–10 °C) with a temperature ramp of 2.5 °C/min (gellan) and 5 °C/min (XLB and agarose) at decreasing temperature.

#### 4.1.3. Swelling Characteristics of Hydrogels

The weights of the hydrogels (four gels per condition; 0%, 0.5% and 1% pea or soy protein concentration with agarose, gellan or XLB) were measured at days 0, 1, 2, 3 and 7. The preparation of the gels and the weighing were carried out under sterile conditions to prevent possible contamination. The initial weight for all gels was taken 15 min after adding 1 mL medium (DMEMhg with 10% fetal bovine serum, 1% stable L-glutamine (No. P04-82050, PanBiotech, Aidenbach, Germany) and 0.2% Primocin^®^ (No. Ant-pm-2, InvivoGen, San Diego, CA, USA). After weighing the gels on day 3, the medium was completely replaced with fresh medium. Additionally, four gels were dried at 60 °C for seven days in an oven for dry weight determination.

The swelling ratio was calculated with the following formula:

$$\mathrm{Swelling}\;\mathrm{ratio}\;\left[\%\right]=\frac{\mathrm{initial}\;\mathrm{wet}\;\mathrm{weight}-\mathrm{dry}\;\mathrm{weight}}{\mathrm{dry}\;\mathrm{weight}}*100$$

### 4.2. Cell Culture

The murine myoblast C2C12 cell line was obtained from CLS Cell Lines Service GmbH (No. 400476, Eppelheim, Germany). 5 × 10^3^ C2C12 cells/cm^2^ were cultured in DMEM high glucose containing 10% fetal bovine serum, 1% stable L-glutamine and 0.2% Primocin (culture medium) at 37 °C with 5% CO_2_. At a confluence of around 70% the cells were washed once with PBS with calcium and magnesium (PBS+; No. 17-513F, Lonza group, Basel, Switzerland) and passaged using 0.05% trypsin (No. 25200-072, Gibco^TM^ Thermo Fisher Scientific) diluted in 0.53 mM EDTA solution (5 M EDTA stock solution (No. 11568896, Fisher Scientific GmbH)) diluted in PBS without calcium and magnesium (PBS-; No. BE17-516F, Lonza group). After 3–4 min at 37 °C with 5% CO_2,_ the incubation was stopped by adding culture medium. A total of 1.5 × 10^4^ cells were seeded per well of a 48-well plate (No. C-8219, neoLab Migge GmbH, Heidelberg, Germany). Phenol red free DMEM high glucose (No. P04-01161, PanBiotech) was used with 10% FCS, 1% stable L-glutamine and 0.2% Primocin for cell toxicity analysis and cell-laden gels.

### 4.3. Biocompatibility Analysis

#### 4.3.1. Indirect Cell Toxicity Test

An indirect cytotoxicity test was performed based on the ISO-10993-5. In brief, one-day and three-day leachates of 15 different biomaterial combinations (0%, 0.5% and 1% pea or soy protein concentration with agarose, gellan or XLB) were produced and given to the cells. For this, 1 mL of each warmed biomaterial solution was poured into wells of a 24-well plate and cooled down at RT. For gellan and XLB gels, 1 mL culture medium (s. 4.2) for crosslinking was added for 15 min. After the gelation process was completed, the gel and the medium were transferred into a 50 mL centrifugation tube (No. T2318, Greiner Bio-One GmbH). All gels were covered with 4 mL culture medium in total (gel-medium ratio 1:4) and then put into an incubator with 5% CO_2_ and 37 °C for 1 day or 3 days. As a negative control, a pure medium was incubated at 37 °C and 5% CO_2_. For the positive control, Triton^TM^ X-100 (No. T8787, Sigma-Aldrich, St. Louis, MI, USA) was added to a total volume of 0.1% to the previously incubated medium. After removing the cell culture medium, the leachates and controls were added to the wells (48-well plate) that were seeded with 1.5 × 10^4^ C2C12 cells/well the day before. After one day of incubation at 37 °C and 5% CO_2_, the cells were analyzed. Three wells were each analyzed with LDH and resazurin assay, and one well was used for Live/Dead-staining.

#### 4.3.2. Formation of Cell-Laden Gels

The cell-laden gels were prepared similarly as described above (s. 4.1.1 Preparation of Gels). Instead of using medium alone, the same volume (0.15 mL) of medium was supplemented with 4.95 × 10^6^ C2C12 cells, resulting in a final concentration of 3 × 10^6^ cells/mL gel and therefore 3 × 10^5^ cells/gel. Lastly, the cell suspension was added to the polysaccharide-protein solution and heated at 37 °C. Again, 100 µL gels were manufactured by filling the volume into an in-house scaffold using a syringe. After cooling down at RT, the hydrogels were covered with 1 mL phenol red-free medium and the scaffolds were removed after a further 15 min.

#### 4.3.3. LDH Assay

To measure the released amount of lactate dehydrogenase (LDH) in cell culture supernatants, the LDH Cytotoxicity Detection Kit from Takara Bio Inc. (No. MK401, Kusatsu, Japan) was used. Briefly, duplicates of 50 µL supernatant of each well were pipetted into a 96-well plate (No. 655 180, Greiner Bio-One GmbH) and mixed with 50 µL reaction solution (catalysator:dye solution 1:50) and incubated for 30 min in the dark at RT. At the end, the absorbance at 490 nm and a reference wavelength at 680 nm were measured with a SpectraMax^®^ iD3 microplate reader (Molecular Devices LLC, San José, CA, USA).

#### 4.3.4. Resazurin Assay

To determine the metabolic cell activity, a resazurin assay was performed. Before usage, the resazurin stock solution (0.11 mg/mL resazurin (No. R7017, Sigma-Aldrich) in PBS+ was diluted with phenol red free medium (1:100). All medium was removed from the cells and replaced with 350 µL of the resazurin solution per well (48-well plate). After 4 h of incubation at 37 °C and 5% CO_2_ in an incubator, triplicates of 100 µL of the metabolized resazurin solution were pipetted into a 96-well plate and measured at a SpectraMax^®^ iD3 microplate reader (absorbance: 570 nm, reference wavelength: 600 nm).

#### 4.3.5. Live/Dead-Staining

A Live/Dead^TM^ viability/cytotoxicity kit for mammalian cells from Invitrogen by Thermo Fisher Scientific (No. L3224) was used for Live/Dead-staining. The staining solution with ethidium homodimer-1 (1:500), calcein AM (1:2000) and Hoechst 33342 (1:1000, No. 4082S, Cell Signaling Technology, Danvers, MA, USA) was prepared with PBS+. The supernatant was carefully removed from the cells/gels. Afterwards, 200 µL (per 48 well) or 300 µL (per 3D hydrogel) staining solution was added. After 30 min (2D cell cultures) or 60 min (3D hydrogels) incubation in the dark at RT, the cells were microscoped with 1:10 magnification with a fluorescence microscope (Axio Observer, Carl Zeiss AG, Jena, Germany). Different fluorescence channels were used for each staining reagent (desoxyribonucleic acid (DNA) staining with Hoechst 33342 in blue (460 nm), viable cell staining with calcein in green (550 nm) and dead cell staining with ethidium homodimer-1 in red (600 nm).

### 4.4. Statistical Analysis

The experiments were done in triplicate or quadruplicate, as mentioned. The results are presented as means ± standard deviations. The statistical significance was determined using a two-sided t student test with *p* < 0.05 considered as significant.

## Figures and Tables

**Table 1:** Food and environmental characteristics of the biomaterials agarose, gellan, xanthan, locust bean gum, soy and pea protein.

	Biomaterial	Taste	Maximum Daily Intake	Allergy Risks	Source/Origin	Regulations as Food	Usage in Food Industry	Annual Production	Environmental Impact
**Hydrogel**	**Agarose/Agar**	Tasteless [31]	64 mg/kg body weight per day [32]	Very low [32]	Sea weed, red algae [23]	Evaluated as food additive [32]	Thickener, stabiliser, gelling agent [31]	>20,000 t [33]	Low [34]
	**Gellan**	Tasteless [35]	200 mg/kg body weight per day [36]	Very low [36]	Pseudomonas bacteria [37]	Evaluated as food additive [36]	Thickener, gelling agent and stabiliser [31]	>10,000 t [38]	Low-medium [39,40,41]
	**Xanthan**	Tasteless [42]	214 mg/kg body weight per day [43]	Very low [43]	Xanthomonas [44]	Evaluated as food additive [43]	Thickener, stabiliser, emulsifier, foaming agent [31]	>30,000 t [45]	Low-medium [46,47]
	**Locust** **Bean gum**	Tasteless, Risk of leguminous taste when heated [48,49]	500 mg/kg body weight per day [50]	Very low [50]	Carob tree seeds [51]	Evaluated as food additive [50]	Thickener, gelling agent [31]	>10,000 t [52]	Low [53,54]
**Additive**	**Pea protein**	Untreated: Bitter, beany, green, grassy, and leafy [55,56]	30 g per day [57]	Low [58]	Pea, Pisum sativum [59]	Evaluated as food additive [60]	Emulsifier, foaming agent, gelling agent [56]	>200,000 t [61]	Medium [62]
	**Soy protein**	Untreated: Bitter, beany, fatty, green [55]	25–100 g per day [63]	Low [64]	Soybean, Glycine max [65]	Evaluated as food additive [66]	Emulsifier, foaming agent, gelling agent, fat and water absorption [67]	>1,000,000 t [65]	Low-medium [62,68,69]

**Table 2:** Summarized results for different polysaccharides (agarose, gellan and xanthan-locust bean gum blend) with application relevant information: (+) very good properties, (+/o) good properties, (+/−) acceptable properties, and (−) poor properties.

Polysaccharide	Gel Formation with Protein	Form Stability over Time	Gelation Temperature	Biocompatibility	Encapsulation of Cells
**Agarose**	uniform gels > 2.5% protein less uniform < 7.5% protein	no weight change	23–24 °C with and without protein	non toxic	possible, but with inhomogeneous cell distribution
	**(+)**	**(+)**	**(+)**	**(+)**	**(+/−)**
**Gellan**	uniform gels > 1% protein less uniform gels < 7.5% protein	slight weight change	29.4 °C without protein Less with protein	non toxic	possible with homogeneous cell distribution
	**(+/−)**	**(+/o)**	**(+)**	**(+)**	**(+)**
**XLB**	uniform gels > 1% protein	significant weight change	56 °C without protein Less with soy protein	non toxic	not possible
	**(+/−)**	**(−)**	**(−)**	**(+)**	**(−)**

**Table 3:** Mixing ratios of agarose, gellan and XLB hydrogels with different protein concentrations of SPI and PPI.

Protein Concentration [%]	2% Agarose, 2% Gellan or 1% XLB [µL]	12% SPI [µL]	Distilled H_2_O [µL]	15% PPI [µL]	Distilled H_2_O [µL]	2% SPI or PPI [µL]	Distilled H_2_O [µL]
0	750	0	750	0	750	0	750
0.5	750	62.5	687.5	50	700	375	375
1	750	125	625	100	650	750	750
2.5	750	312	438	250	500	-	-
5	750	625	125	500	250	-	-
6	750	750	0	-	-	-	-
7.5	750	-	-	750	0	-	-

## Data Availability

Not applicable.

## Supplementary Materials

The following are available online at https://www.mdpi.com/article/10.3390/gels8020094/s1, Figure S1: Sol-gel transition of polysaccharides (agarose, gellan and XLB) blended with 0%, 0.5% and 1% pea or soy protein with elastic and viscous modulus and phase angle versus temperature (1.0 Hz frequency and 1 Pa shear stress). With gellan, a shift of the gelation point to lower temperatures of the blends compared to the native gel can be observed. Figure S2: XLB-protein blends (0%, 0.5% and 1% pea or soy protein) mixed with 3 × 10^5^ C2C12 cells/gel in plate (24 well) one day after gel preparation. Scale bar: 1.55 cm.
