# Supplementary figures and images for: Scaffolds for Cultured Meat on the Basis of Polysaccharide Hydrogels Enriched with Plant-Based Proteins
Source: Gels. 2022 Feb 4;8(2):94. doi: 10.3390/gels8020094 (PMC8871916; doi:10.3390/gels8020094)

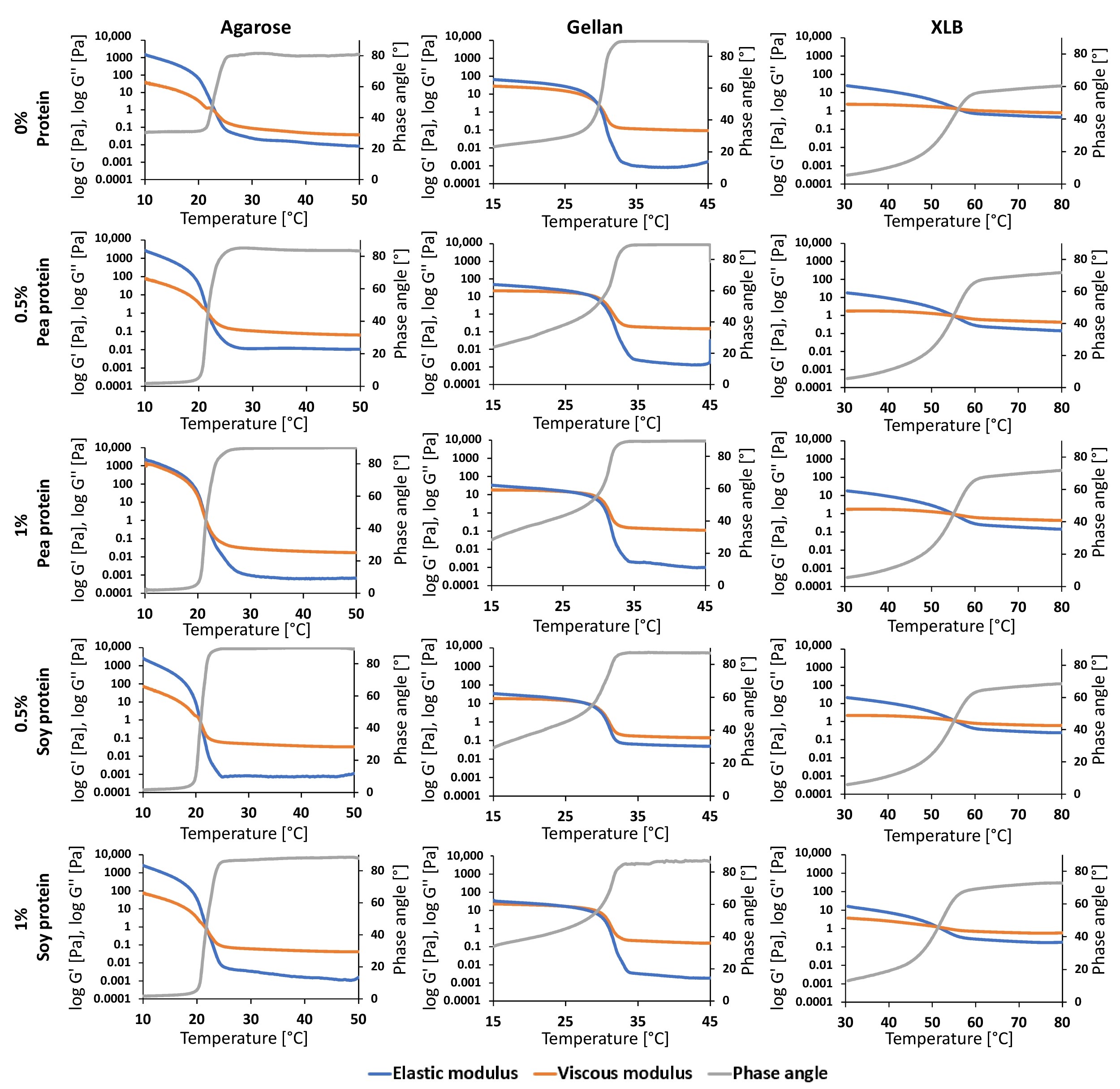

Supplement: Supplementary file 1 [file gels-08-00094-s001.zip › Figure S1_Sol-gel transition curves_2.jpg]

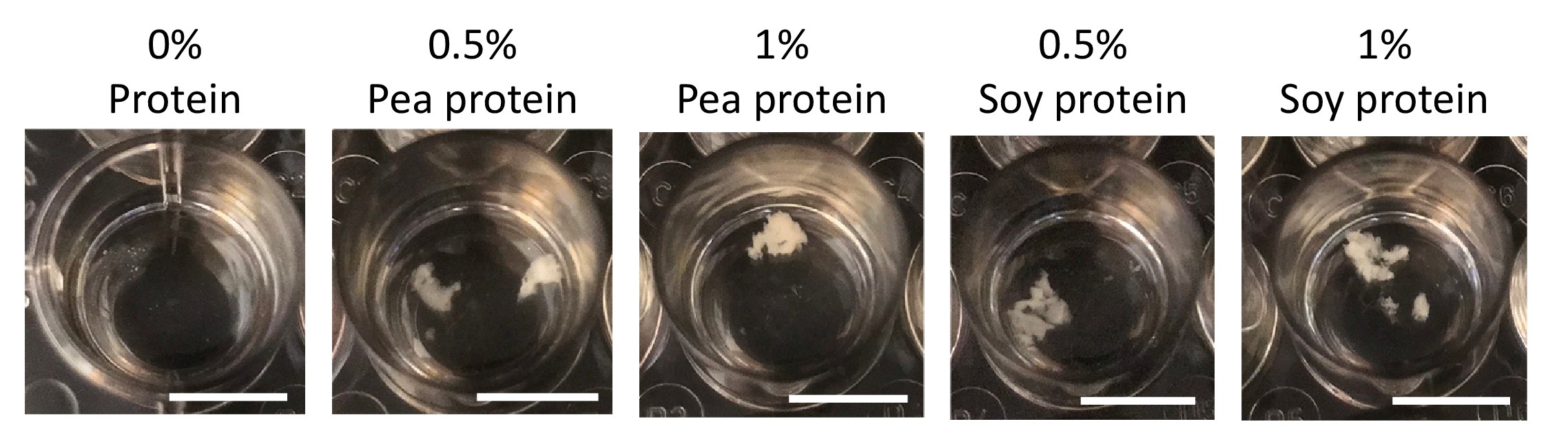

Supplement: Supplementary file 1 [file gels-08-00094-s001.zip › Figure S2_Macroscopic cell laden XLB gels.jpg]
